# Factors Affecting the Implementation of Community Perinatal Midwifery Care in Greece, in Relation to the Perspectives, Attitudes, and Expectations of Primary Healthcare Midwives

**DOI:** 10.7759/cureus.80566

**Published:** 2025-03-14

**Authors:** Maria Kontoyannis, Maria Dagla, Eriketi Kokkosi, Victoria Vivilaki, Christos Katsetos, Angeliki Sarella

**Affiliations:** 1 Department of Midwifery, Faculty of Health and Caring Sciences, University of West Attica, Athens, GRC; 2 Department of Obstetrics and Gynaecology, Tzaneio Hospital, Piraeus, GRC

**Keywords:** community midwives, labor and birth care, midwifery care, midwives' attitudes and perspectives, midwives' expectations, midwives' views, perinatal care, post-natal care, pregnancy care

## Abstract

The implementation of community perinatal midwifery care in Greece, as in many other healthcare settings, is influenced by a variety of factors that interconnect, among others, with the perspectives, attitudes, and expectations of primary healthcare midwives. Understanding these factors is crucial for improving maternal and neonatal standards of care and ensuring that midwives are successfully reinforced in their professional roles. A comprehensive keyword search of the literature was conducted on five databases to identify recent studies: Cumulative Index of Nursing and Allied Health Literature (CINAHL), Medline, PubMed, Scopus, and Google Scholar. The search was restricted to articles published between 2008 and 2024, and search terms and variations of search terms included but were not limited to "community midwives", "community midwifery", "views of", "expectations", "perspectives of community midwives", and "implementation of community midwifery". As opposed to local reality, there seems to be an extensive global variety of literature associated with the views and expectations of midwives regarding their public role in the community. The fruitful implementation of community perinatal midwifery care in Greece depends on the merging of various factors, including the healthcare system's structure, support from the public and governmental policy, cultural attitudes toward birth, and the educational background of midwives. Community midwives' views and expectations play an essential role in how they navigate various challenges and opportunities they face along their daily professional life. To develop the delivery of community-based perinatal care, it is vital to provide a multilayered approach that contains structured policy improvements, increased assistance for midwives, enhanced public education, and interprofessional teamwork.

## Introduction and background

The implementation of community perinatal midwifery care through the years in Greece has delivered great achievement in terms of improving maternal and child health outcomes [1,2], but it has faced some challenges. This review aims to explore the perspectives, attitudes, and expectations of primary healthcare midwives (community midwives) regarding the factors influencing the implementation of the community perinatal midwifery care legislation in Greece. In order to develop effective policies to overcome obstacles and certify successful implementation of the current legislation relating to perinatal midwifery community care, it is essential to comprehend these factors.

This review draws upon the theoretical framework of social dynamics that highlight the social and cultural factors shaping individuals' beliefs, attitudes, and behaviors. Social dynamics assist in understanding how primary healthcare midwives' perspectives on community perinatal midwifery care are influenced by societal norms, cultural values, and professional socialization.

The second theoretical framework that applies to this review is the distribution of innovation concept. This theory explains how innovations are accepted by individuals and organizations over time. It will be used to analyze the factors that influence the approval of community care legislation among primary healthcare midwives, including perceived relative benefits, compatibility, complexity, and observability.

## Review

A comprehensive keyword search of the literature was conducted on five databases to identify recent studies: Cumulative Index of Nursing and Allied Health Literature (CINAHL), Medline, PubMed, Scopus, and Google Scholar. The search was restricted to articles published between 2008 and 2022, and search terms and variations of search terms included but were not limited to "community midwives", "community midwifery", "views of", "expectations", "perspectives of community midwives", and "implementation of community midwifery".

International background

There is vast research on community perinatal midwifery care implementation, where a significant body of research has been conducted on the implementation of community perinatal midwifery care in various countries around the world [2-8]. These studies have identified a range of factors that influence the success or failure of community perinatal midwifery care programs.

Some of the key factors that have been found to be significant include government support that is essential for the successful implementation of community perinatal midwifery care. This, among others, comprises financial investments, suitable policy development, and regulatory oversight. Furthermore, well-trained and qualified midwives are important for providing high-quality community perinatal midwifery care. Midwifery education programs should be adequately funded and aligned with the needs of community perinatal midwifery care programs. Effective collaboration between midwives and other healthcare providers, such as obstetricians, pediatricians, and nurses, is crucial for ensuring the safety and quality of community perinatal midwifery care. Community engagement is essential for the success of community perinatal midwifery care programs. These programs should be developed in collaboration with local community groups and should be responsive to their needs and preferences. Cultural and social factors can significantly influence the acceptability and adoption of community perinatal midwifery care programs. These programs should be socially sensitive and should address the precise needs and beliefs of the target population [7,8].

A number of factors seem to influence primary health community midwives' viewpoints. More specifically, community midwives' perspectives on community perinatal midwifery care, are formed by a range of factors [3,9,10,11], including community midwives' own beliefs and values about childbirth, women's health, and the role of midwives can have a serious impact on their attitudes towards community perinatal midwifery care [12]. Previous experiences with childbirth and midwifery care can influence their perspectives on community perinatal midwifery care. Community midwives’ education and training in midwifery shape their knowledge and skills related to community perinatal midwifery care. The culture of the healthcare organization where primary health care midwives work can have an impact on their attitudes toward community perinatal midwifery care.

For example, organizations that are supportive of midwifery and community perinatal midwifery care are more likely to have community midwives who are positive about this specific approach. Interactions with colleagues and peers influence their views towards community perinatal midwifery care. Positive experiences and support from colleagues can encourage community midwives to adopt a friendlier community perinatal midwifery care attitude [9,10]. 

It is important to understand that these factors are not equally exclusive and can relate in complex ways to influence community midwives' perspectives on community midwifery care. Comprehending these factors can assist in the identification of strategies for promoting the acceptance of community perinatal midwifery care among community midwives [13].

Table 1 summarizes perinatal midwifery care in Greece compared to the UK, USA, Canada, and Sweden.

**Table 1:** Comparative table summarizing perinatal midwifery care in Greece compared to the UK, USA, Canada and Sweden.

Aspect	Greece	UK	USA	Canada	Sweden
Midwifery model	Obstetric-led care dominates; midwifery limited	Midwifery-led care with NHS support	Obstetric-led care dominates; midwives less common	Midwifery-led care integrated with healthcare	Strong midwifery-led model; high home birth rates
Place of birth	Mostly hospitals; home births rare	Hospitals, birth centers, home births	Predominantly hospitals; birth centers limited	Hospitals, birth centers, home births supported	Hospitals, birth centers, high home birth rates
Access to midwives	Limited, mostly in public hospitals	Freely available through the NHS	Expensive private practice midwives	Covered under the public health system	Universal midwifery access
Cost of care	Public sector: free; private sector: costly	Free under the NHS	High costs; insurance-dependent	Free or low-cost through public system	Free under public healthcare system
C-section rate	55% (highest in Europe).	26%	32%	28%	17% (one of the lowest)
Midwifery autonomy	Limited autonomy; obstetricians oversee care	Independent midwives available	Limited autonomy; dependent on OB-GYNs	Autonomous midwives in hospitals and communities	Highly autonomous midwifery profession
Postnatal care	Minimal follow-up after birth	Home visits up to 10 days post-birth	Follow-ups depend on insurance coverage	Regular home visits and check-ups	Comprehensive postnatal care for months

Historical and cultural background in the Greek context

The historical growth of midwifery in Greece is deeply intertwined with the country's broader social and cultural context as in any other country [1,2]. The role of midwives has progressed over time, reflecting changes in societal attitudes toward women's health, childbirth, and family planning. In the past few years, and up until recently, midwives played a central role in providing care for women during pregnancy, childbirth, and the postpartum period. However, with the rise of modern medicine and the establishment of hospital-based obstetric care, the role of midwives gradually weakened [1].

In recent decades, there has been a rising acknowledgement of the status of midwifery care in Greece, mainly in the context of improving maternal and child health outcomes. This recognition has been influenced by several factors, including increasing rates of caesarean section [1,2]. The high rates of caesarean section in Greece have raised concerns about the quality of care provided to women during childbirth. Midwifery care, with its emphasis on natural childbirth and low-intervention approaches, has been seen as a potential solution to this problem. There has also been a growing demand for personalized care, as women in Greece are increasingly seeking more tailored ways of care that are based on evidence-based information provision and a more autonomous approach regarding their care during pregnancy and childbirth. Midwives, with their woman-centered philosophy of care, are well-equipped to provide this type of care [13]. 

Regarding health system factors, the structure and functioning of the Greek healthcare system play a key role in the implementation of community perinatal midwifery care [13,14], as the national healthcare system is currently undergoing reforms aimed at decentralizing healthcare services and increasing access to primary care. These reforms present opportunities and challenges for the implementation of community perinatal midwifery care by way of reorganizing the healthcare services, so this can eventually produce a more favourable environment for the development of community-based midwifery programs. By shifting the focus of care to the primary care level, it is possible to increase access to midwifery services and reduce the burden on hospitals.

On the other hand, the implementation of community perinatal midwifery care may face challenges due to the existing infrastructure and resources within the Greek healthcare system [13]. For example, there may be a need to invest in further training and education for midwives of the community, as well as in the development of appropriate facilities and equipment for providing midwifery care in community settings.

As far as the issue of viewpoints of primary healthcare midwives on the role of community perinatal midwifery care in maternal and child health concern, it seems that midwives in the community may view community perinatal midwifery care as offering several benefits compared to hospital-based care [6], including increased continuity of care through community perinatal midwifery care that allows midwives to provide consistent care throughout the pregnancy, childbirth, and postpartum period, promoting a solid relationship with the woman and her family. Community perinatal midwifery care highlights and focuses on natural childbirth and low-intervention approaches, which can lead to improved maternal and child health outcomes. Midwives can tailor their care to the specific needs and preferences of each woman, taking into account her personal beliefs and her cultural background. Community perinatal midwifery, through personalized care, can help to reduce the rate of unnecessary interventions, such as caesarean sections and episiotomies, which can have negative consequences for both mother and baby [10].

Furthermore, the views of community midwives on the potential impact of community perinatal midwifery care on maternal and child health outcomes include [11] reduced maternal mortality and morbidity, such as postpartum hemorrhage, infection, and preeclampsia, and better birth outcomes, such as lower rates of preterm birth, low birth weight, and infant mortality. Community perinatal midwifery care, according to community midwives’ viewpoints, can promote stronger maternal-child bonding by providing a supportive and empowering environment for childbirth, contribute to improving women's overall health and well-being as this improves maternal and child health and women's overall health and well-being, and reduce the risk of postpartum depression and anxiety by providing a supportive and caring environment. Community perinatal midwifery care, according to midwives’ views, can improve women's overall quality of life by promoting positive birth experiences and fostering healthy relationships with their babies and partners. As a result, community perinatal midwifery care assists women in feeling more empowered and in control of their own health and childbirth experiences [12].

Midwives in the community may believe that the successful implementation of community perinatal midwifery care requires adequate funding, staffing, and infrastructure [4]. Sufficient funding to support the training, education, and ongoing operation of community perinatal midwifery care programs. In addition, adequate numbers of qualified community midwives are important in order to meet the needs of the population. Appropriate facilities and equipment for providing midwifery care in community settings, including birthing centers, home visit services, and telemedicine capabilities. Community midwives may expect to obtain complete training and continuing education to prepare them for their new role in community perinatal midwifery care. This may include clinical skills, by training in essential midwifery skills, such as conducting prenatal and postpartum examinations, assisting with childbirth, and managing common complications. This can be improved through evidence-based practice, education that is based on the latest research, and evidence-based guidelines related to community perinatal midwifery care [13].

According to the literature [14,15], community midwives believe that inter-professional partnership should be enhanced through training on ways of successful collaboration with other healthcare providers, such as obstetricians, pediatricians, and nurses. In addition, midwives in the community may expect to receive ongoing support and mentorship from other experienced midwives and healthcare administrators by means of supervision and guidance from experienced midwives, in order to ensure that quality of care is provided so to be able to address any challenges. Opportunities for mentorship and professional development are important in order to support community midwives in their career growth, as well as prospects for collaboration with other healthcare providers and community organizations to address the needs of women and their families.

According to research [3], factors that influence community midwives’ perspectives, attitudes, and expectations include, lack or availability of human resources, information management, continuity of care, cultural barriers within a country, legal and managerial barriers, the presence or not of care facilities mainly in rural areas, medical equipment, and geographical access. In addition, midwives' views are influenced by traditional values regarding midwives’ professional status in the community, insufficient leadership, deprived work environments, and a low level of qualified responsibility, which hinder midwives’ ability to provide respectful maternity care.

Further significant issues influencing midwives' perspectives, attitudes, and expectations include the presence of suitable maternity service strategies, communication of ongoing changes, interprofessional relationships, and the need for continuity of care within all communities, rural or not. Key factors also include the organization of care, workload concerns, autonomy in practice, continuity of care perceptions, and the need for supportive policies and guidelines for midwife-led models of care [3,13]. More specifically, previous experiences with childbirth and midwifery care, both as patients and as healthcare providers, can significantly shape their perspectives on community perinatal midwifery care. Positive experiences with midwifery care may foster a more favorable attitude toward community perinatal midwifery care, while negative experiences may raise concerns or reservations.

The extent of community midwives’ education and training in midwifery can influence their knowledge and understanding of community perinatal midwifery care principles and practices. Those with more specialized training in midwifery may be more likely to support community perinatal midwifery care and understand its potential benefits [13]. The social and cultural environment in which community midwives are trained and practice can influence their views on community perinatal midwifery care. For example, if midwives in the community are surrounded by a supportive and collaborative environment that values midwifery and community perinatal midwifery care, they may be more likely to adopt positive attitudes toward the approach [16].

Organizational culture, leadership, and support systems

The culture of the healthcare organization where community midwives work can play a significant role in shaping their attitudes towards community perinatal midwifery care. A supportive and inclusive organizational culture that values midwifery and community perinatal midwifery care can create a more favourable environment for the implementation of the approach. Equally important is the issue of a hierarchical and bureaucratic culture, which may pose challenges or obstacles to the adoption of community perinatal midwifery care programs [12]. The support of organizational leaders is crucial for the successful implementation of community perinatal midwifery care. Leaders who are committed to midwifery and community perinatal midwifery care can only deliver the necessary resources, training, and support to community midwives.

The availability of support systems for community midwives, such as mentorship programs, peer support groups, and access to continuing education, can influence their attitudes and behaviors. These systems can provide midwives in the community with the necessary guidance, encouragement, and professional development opportunities to support their work in community perinatal midwifery care [12]. 

Mentorship programs offer structured guidance from experienced midwives to those who are newer to the profession or transitioning into community-based practice. These programs help midwives navigate challenges such as complex cases, home birth logistics, and community engagement. Having a mentor can foster confidence, enhance problem-solving skills, and reinforce best practices, ultimately improving patient outcomes. In addition, mentorship fosters a sense of belonging and professional identity, reducing feelings of isolation that community midwives may experience. In addition, peer support groups provide a space for midwives to share experiences, discuss challenges, and offer emotional support to one another. Community midwifery can be demanding, with unpredictable hours and emotionally intense situations. Engaging with peers allows midwives to debrief, share coping strategies, and exchange knowledge on clinical and non-clinical aspects of care. This form of support can help mitigate stress and burnout, fostering resilience and job satisfaction.

Broader societal factors

Cultural norms and values related to childbirth, women's health, and the role of healthcare providers may influence community midwives’ perspectives on community perinatal midwifery care. For example, in cultures where childbirth is viewed as a private and personal event, community midwives may be more likely to support a model of care that emphasizes continuity and individualized support [17].

In some cultures, childbirth is seen as an intimate and family-centered experience rather than a medical event. In such societies, there is often a strong preference for home births or birthing centres where women can have greater control over their birthing experiences. Community midwives working within these cultural contexts are more likely to support care models that emphasize continuity, holistic support, and respect for traditional birthing practices. They may also place a high value on building trusting relationships with expectant mothers and their families, ensuring that care remains personalized and non-intrusive [17,18].

Societal expectations regarding gender roles and the division of labour within families can impact community midwives’ attitudes towards community perinatal midwifery care. In cultures where women are expected to be primary caregivers for their children, community midwives may be more likely to support a model of care that emphasizes home births and community-based support. Economic factors can also influence community midwives’ perspectives on community perinatal midwifery care. In regions with limited access to healthcare resources, community midwives may be more likely to support a model of care that is affordable and accessible to all women [18].

## Conclusions

This literature review will contribute to a better understanding of the factors influencing the implementation of community perinatal midwifery care in Greece. By exploring the perspectives, attitudes, and expectations of local community midwives, the study will provide valuable insights for policymakers and healthcare administrators to develop effective strategies for promoting the adoption and successful implementation of community perinatal midwifery care.

The insights gained from this review will be instrumental for policymakers and healthcare administrators in designing evidence-based strategies that support midwives in their transition to community-based care. These strategies may include policy reforms, enhanced training programs, interdisciplinary collaboration, and the integration of midwifery-led models within the broader healthcare system. By identifying key areas for intervention, this study aims to contribute to the development of a sustainable framework that ensures equitable access to comprehensive, patient-centered perinatal care for mothers and newborns across Greece.
